# Glomerulopathy associated with schistosomiasis mansoni

**DOI:** 10.1590/2175-8239-JBN-2025-0084en

**Published:** 2025-10-06

**Authors:** Irma Bandeira de Sousa Pontes, Amaro Nunes Duarte, Cristiane Bitencourt Dias

**Affiliations:** 1 Universidade de São Paulo, Faculdade de Medicina, Hospital das Clínicas, Laboratório de Fisiopatologia Renal, São Paulo, SP, Brazil.; 2 Universidade de São Paulo, Faculdade de Medicina, Departamento de Patologia, São Paulo, SP, Brazil.

**Keywords:** Schistosomiasis, Glomerulonephritis, Membranoproliferative, Immunosuppresive Agents, Biomarkers, Antigens

## Abstract

Schistosomiasis is a parasitic disease directly related to poor sanitation. In Brazil, 42 million people live in areas where the disease is endemic, and it is still considered a serious public health problem. The kidney is one of the organs typically affected by this worm, with descriptions of renal tubular alterations, acute kidney injury and glomerular diseases. Among patients with active schistosomiasis, the incidence of glomerulopathy is 5–6% overall and 15% in those with the hepatosplenic form with the most commonly described histological patterns being membranoproliferative glomerulonephritis and focal segmental glomerulosclerosis. Although the pathogenesis of glomerular disease associated with schistosomiasis is uncertain, it is thought to involve immune complexes, especially in membranoproliferative glomerulonephritis, formed against a schistosome antigen and deposited in the glomeruli. The uncertain pathogenesis makes schistosomiasis treatment a challenge, as eradicating the parasite through the use of an anti-parasitic agent does not stop the progression to chronic kidney disease, raising questions about the need for immunosuppressive therapy. Thus, the aim of this review was to describe the so-called classic information on glomerular disease associated with schistosomiasis and to explore its probable pathogenic mechanisms in order to promote future discussion on the development of better treatment options.

## Introduction

Schistosomiasis is a parasitic disease caused by a helminth belonging to the class Trematoda. The disease was first identified by the German physician Theodor Bilharz during an autopsy study of mesenteric veins, conducted in Cairo in 1852; hence the name “bilharziasis” as a synonym for schistosomiasis^1^. In 1908, the Brazilian researcher Manoel Augusto Pirajá da Silva identified *Schistosoma mansoni* as the etiological agent of schistosomiasis in the Brazilian state of Bahia^2^.

The species that cause schistosomiasis in humans include *S*. *haematobium*, found mainly in Africa, *S. japonicum*, endemic to China and other parts of East Asia, and *S. mansoni*, in Latin America. Other species, such as *S. guineensis*, *S. intercalatum*, and *S. mekongi*, also infect humans but have a lower prevalence^3^.

Transmission of Schistosoma sp. occurs when the cercariae—a larval form of the parasite released by freshwater snails—penetrate the skin and mucous membranes during bathing or swimming. The perpetuation of transmission requires infected humans, who excrete helminth eggs in their feces, and aquatic snails, which act as intermediate hosts, releasing infective larvae of the worm into water sources used by humans. After penetrating the human body, the larvae typically affect the kidneys, urinary tract, intestine, and liver^4^.

Kidney involvement in schistosomiasis is variable; the incidence of glomerulopathy is 5–6% among patients with active schistosomiasis and 15% among those with the hepatosplenic form^5^. The most commonly reported histological type is membranoproliferative glomerulonephritis (MPGN)^6,7,8^. Glomerulopathy associated with schistosomiasis has been studied for over half a century, and its multifactorial pathogenesis is not fully understood, making treatment a challenge^9^. Curing schistosomiasis through the use of an antiparasitic agent does not halt the progression to glomerulopathy. Therefore, despite the importance of helminth-derived antigens in the onset of glomerular lesions, other factors, such as autoimmunity and cytokine production, play a role in promoting and worsening the progression to glomerular involvement^9,10^.

Other kidney problems that will not be discussed in the current review, also occur, such as acute kidney injury in 43.3% of patients hospitalized with the hepatosplenic form of schistosomiasis described by Duarte et al.^11^, as well as tubular dysfunction characterized by urinary concentration deficit and urinary acidification deficit, demonstrated by the same authors in another study of patients with the compensated hepatosplenic form^12^. The aim of this review was to describe the so-called classic information on glomerular disease associated with schistosomiasis and to explore its probable pathogenic mechanisms in order to promote future discussion on the development of better treatment options.

## Epidemiology of Schistosomiasis Mansoni-Associated Glomerular Disease

Despite being one of the most prevalent tropical infectious diseases, schistosomiasis is categorized as a neglected tropical disease^13^. According to a statement issued in 2018 by the World Health Organization (WHO), schistosomiasis is second only to malaria on the scale of tropical infectious diseases ranked by importance and socioeconomic impact. It affects almost 240 million people worldwide, and more than 700 million people live in areas where the disease is endemic. It is one of the most widespread parasitic diseases that affect humans^14^. In the Americas, 10 countries and territories are considered endemic for schistosomiasis and 25 million people are at risk^15^.

In Brazil, 42 million people live in areas where schistosomiasis is endemic, and it is still considered a serious national public health problem. The number of reported cases is highest in the northern and northeastern regions of Brazil, despite the implementation of schistosomiasis control programs^16,17^. The kidney is one of the organs typically affected by *S. mansoni*, highlighting here the glomerular diseases associated with this worm. Some histological patterns have been described, but the most frequent are MPGN, focal segmental glomerulosclerosis (FSGS), proliferative mesangial glomerulonephritis, and membranous nephropathy^9,18^.

The histological pattern of the glomerulopathy most often associated with schistosomiasis depends on the clinical presentation of the disease. Hepatosplenic forms are most often associated with MPGN. This was clearly observed in two studies carried out at our institution (Table 1), in which the prevalence of MPGN was found to be 20.8% in the intestinal forms and 70.8% in the hepatosplenic form. There is a predominance of proliferative mesangial glomerulonephritis in the intestinal form, and the FSGS pattern is seen in 25% in both forms of schistosomiasis^9,19^. The idea of a membranous nephropathy associated with schistosomiasis is not well accepted, mainly because it is not included in the African Association of Nephrology (AFRAN) classification. However, two studies carried out in Brazil described this glomerular pattern in a total of 14 cases of schistosomiasis if we consider the two studies^18,19^.

**Table 1:** Demographic and clinical characteristics of patients with schistosomiasis-associated glomerulopathy by presentation form

Characteristic	Abensur et al.^19^	Neves et al.^9^
	(n = 24)	(n = 24)
Age (years), mean ± SD	29.9 ± 12.6	38.58 ± 9.83
Male, n (%)	14 (58.3)	19 (79.1)
Serum creatinine (mg/dL), mean ± SD	1.40 ± 0.7	1.58 ± 0.80
Hypertension, n (%)	11 (45.8)	18 (75.0)
Nephrotic syndrome, n (%)	24 (100)	8 (33.3)
Hepatosplenic form, n (%)	0 (0)	16 (66.6)
Mesangial proliferation, n (%)	8 (33.3)	1 (4.2)
Focal segmental glomerulosclerosis, n (%)	6 (25.0)	6 (25.0)
Membranoproliferative glomerulonephritis, n (%)	5 (20.8)	17 (70.8)
Membranous nephropathy, n (%)	2 (8.3)	0 (0)
Minimal change disease, n (%)	2 (8.3)	0 (0)
Other, n (%)	1 (4.2)	0 (0)

It has been nine years since the WHO published its first roadmap targeting neglected tropical diseases, with goals set for 2020. Notable progress has been made, but several of those goals remain unmet. In response, a new roadmap was developed following a comprehensive global consultation, outlining strategies for the 2021–2030 period. It identifies critical gaps and necessary actions, and sets targets and global milestones to control, eliminate, or eradicate 20 diseases and groups of diseases by 2030, including schistosomiasis^20^.

## Life Cycle of S. Mansoni

Schistosomes are blood flukes that parasitize the venous system of a wide variety of animals, including birds, cattle, and primates^4^. The life cycle of the main schistosomes is quite similar; the differences consist mainly of the intermediate species of snails involved and differences in the distribution of tissues within the definitive hosts^21^. The life cycle of the parasite consists of two phases: the asexual phase, which begins with the penetration of miracidia into the intermediate host and culminates with the elimination of cercariae; and the sexual phase, which occurs in the definitive host^22^. In human schistosomiasis, two hosts are involved^21^: freshwater snails of the genus Biomphalaria (intermediate host) and humans (the definitive host).

Infected humans excrete helminth eggs in their feces, which can contaminate water. The eggs hatch and release ciliated larvae (miracidia) into the water, which penetrate snails, where they undergo structural changes, transforming from primary to secondary sporocysts and producing new larvae (cercariae), which exit the snail and remain in the water until they penetrate human skin^13,17^. The release is greatest when the heat and sunlight are most intense (between 10 a.m. and 4 p.m.). That coincides with the period of the day when the greatest number of people are in the water for bathing or aquatic recreation^2^. The bifurcated larvae (cercariae) penetrate human skin and mucous membranes, losing their tails in the process, transforming into schistosomula, and entering the bloodstream through the capillaries and lymphatic vessels. They are then transported throughout the body by the bloodstream for several days before becoming lodged in the portal vein^2^.

Typically, *S. mansoni* resides in the tributary venules of the portal system, particularly the superior and inferior mesenteric veins, the hemorrhoidal plexus, and the intrahepatic portion of the portal vein. These worms generally migrate within the same vessel or from one vessel to another through anastomoses^13^. In the liver, the juvenile worms feed, grow, and differentiate sexually. They then migrate to the intestine, where they reach adulthood and mate. After mating, the females release eggs, which travel to the intestinal lumen and are excreted in the feces of the host. Within 27 days after penetrating the skin, they transform into adult worms. Egg laying can begin after day 30, and eggs can be found in the host feces from day 40 onwards. The eggs continue to be eliminated in human feces for an average of five years, although there are reports of patients eliminating eggs for up to 30 years after leaving an endemic area^23^.

## Clinical Picture

### Immediate Manifestations

The innate immune response to dying or dead larvae in the skin where cercariae penetrate gives rise to hypersensitivity reactions^3^ and can cause a maculopapular pruritic rash that varies in size from 1 to 3 cm, known as cercarial dermatitis or swimmer’s itch^24,25^. During the passage through the epidermis and dermis, an immediate hypersensitivity reaction occurs, with activation of several components of the innate immune response. Within two days, an infiltrate of polymorphonuclear, mononuclear, and Langerhans cells occurs, together with local production of macrophage inflammatory protein (CCL3), interleukin (IL)-1b, IL-6, IL-12p40, and IL-10. After four to five days, this scenario is still predominant, with an influx of CD4+ T lymphocytes and greater production of IL-12p40, as well as production of interferon gamma and IL-4, all of which decrease in the second week^26^. Cercarial dermatitis usually occurs in individuals exposed to Schistosoma sp. for the first time, such as travelers and migrants to an area endemic for schistosomiasis^3^.

### Acute Schistosomiasis

After successful cercarial penetration and schistosomula formation, infection with Schistosoma sp. can proceed to an acute stage, also known as Katayama fever, named after a district of Hiroshima, Japan, where *S. japonicum* was first detected in a human^3^. A history of contact with contaminated water (14–84 days before symptom onset) is common^25^.

The symptoms of acute schistosomiasis are caused by systemic hypersensitivity reactions and formation of immune complexes in response to antigens released during schistosomula migration or the initiation of egg deposition^3^. Such symptoms include fever, headache, generalized myalgia, right upper quadrant pain, and bloody diarrhea. Respiratory symptoms have been reported in up to 70% of individuals infected with *S. mansoni*, and there can be radiological evidence of interstitial pneumonitis. Tender hepatomegaly is usually present, and splenomegaly occurs in a third of cases. In rare cases, aseptic meningitis develops. At this stage, all patients have eosinophilia and most test positive for schistosomiasis on serologic tests^25^.

Acute schistosomiasis is rarely observed in people living in areas where the disease is endemic^27^. This lack of susceptibility to acute symptoms might be due to in utero desensitization, resulting in a reduced immune response to schistosome antigens in infants born to infected mothers or to repeated exposure to cercariae that induces IL-10 production by CD4+ T cells in the skin, resulting in a regulatory immune response^3^.

### Chronic Schistosomiasis

Chronic schistosomiasis is mainly due to a granulomatous inflammatory reaction against schistosome eggs deposited in various organs and tissues^28^. The eggs actively secrete antigenic glycoproteins, which facilitate their passage through blood vessels, where they are deposited^3^. The intensity and duration of the infection determines the amount of antigen released and the severity of chronic fibrosis/obstruction^25^. Some eggs remain in the capillaries of the intestinal mucosa, while others are carried by the mesenteric circulation to the liver, where they become lodged in the hepatic sinusoids. The release of soluble antigens from the eggs induces the mobilization of macrophages, eosinophils, lymphocytes, and plasma cells, a process that is mediated by tumor necrosis factor alpha, as well as by CD4+, Th1, Th2, and CD8+ T lymphocytes. Macrophages come into contact with the egg, forming multinucleated syncytial masses; some of those cells transform into fibroblasts, guiding the organization of concentric layers throughout the granuloma, with extensive collagen production^26^. Periovular granulomas are large, with predominantly necrotic and exudative characteristics, and appear as translucent granules disseminated on the serosal surfaces of the liver and intestines^24^. The high proliferative response of lymphocytes induced by soluble egg antigens at this stage of the infection progressively diminish as the infection becomes chronic^3^.

Chronic schistosomiasis can have a polymorphic presentation, including intestinal, hepatointestinal, and hepatosplenic forms, pulmonary vascular disorders, pseudotumoral forms, schistosomal nephropathy, and ectopic lesions^26^. The hepatointestinal form is nothing more than the intestinal form associated with hepatomegaly. The pure intestinal form is uncommon. It may be asymptomatic or accompanied by intermittent abdominal pain, discomfort, loss of appetite and, in some cases, bloody diarrhea, in which the patient has a low worm load^3,28^. In the hepatosplenic presentation, there is granuloma formation caused by the deposition of eggs in the liver, resulting in periportal fibrosis^28^. Periportal fibrosis is usually observed in adults and, sometimes, in adolescents living in areas with very high transmission of schistosomiasis. The severity of periportal fibrosis correlates with the intensity and duration of the infection. Occlusion of the portal branch as a result of periportal fibrosis can lead to marked portal hypertension, often accompanied by severe enlargement and induration of the spleen. In some cases, portal hypertension leads to the development of esophageal varices, which can rupture and are associates with a high risk of fatal hematemesis. Other complications include ascites, growth retardation, and severe anemia. However, in contrast to liver cirrhosis of alcoholic or other etiologies, in schistosomiasis, even with marked periportal fibrosis and portal hypertension, hepatocyte damage is not observed and liver enzymes remain normal. One of the most serious clinical outcomes of schistosomiasis is neuroschistosomiasis, which is caused by the inflammatory response around the eggs in the cerebral or spinal venous plexus^3^.

Pulmonary schistosomiasis is caused by portal caval shunting, in which venous blood bypasses the liver through collateral veins connecting the portal vein to the superior vena cava, and eggs are transported to the pulmonary capillaries, where they induce granulomas in the perialveolar area. Those granulomas can lead to fibrosis, resulting in pulmonary hypertension and cor pulmonale^3^.

Patients with schistosomiasis who have glomerular involvement can be asymptomatic, a fact that contributes to the complexity of estimating the true incidence of this condition, given that glomerular involvement can also be self-limited^29^. Patients with schistosomiasis have nephrotic syndrome, isolated subnephrotic proteinuria, acute glomerulonephritis, mixed syndrome, or chronic kidney disease^9,19^.

In schistosomal glomerulopathy, the pathophysiological mechanism is distinct from those of other clinical forms of schistosomiasis, in which the formation of granulomas around the eggs is the most important event. In glomerulopathy, the antigen-antibody reaction mediated by the formation of immune complexes deposited in the glomeruli represents the probable pathogenic mechanism of the renal involvement^26^. The pathogenic mechanisms of glomerulopathy are addressed below.

## Glomerular Histological Patterns

The most frequently reported histological types of schistosomal glomerulopathy, MPGN and FSGS, have mixed syndrome and nephrotic syndrome as their main clinical presentations, respectively^9,19^. The diversity of kidney histological lesions already found led to a clinical-pathological classification of schistosomal glomerulopathy, endorsed in 1992 by the AFRAN, which recognized six categories of glomerular diseases^4,5^, as detailed in Chart 1.

**Chart 1:** Histological classification according to the african association of nephrology

Class	Renal histology	Clinical and laboratory presentation
I	Mesangial proliferative glomerulonephritis	Microhematuria and subnephrotic proteinuria
II	Diffuse exudative glomerulonephritis	Acute nephritic syndrome
III	Membranoproliferative glomerulonephritis	Mixed syndrome and hypertension
IV	Focal segmental glomerulosclerosis	Nephrotic syndrome and hypertension
V	Amyloidosis	Nephrotic syndrome
VI	Cryoglobulinemic glomerulonephritis	Purpura, vasculitis, arthritis, hypertension, and mixed syndrome

In the AFRAN classification, class II glomerulopathy applies to cases in which there is salmonellosis and the clinical picture resembles that seen in acute diffuse glomerulonephritis occurring after staphylococcal/streptococcal infection, including consumption of complement C3 only. In class V glomerulopathy, amyloidosis is due to the deposition of amyloid A protein and can also occur in association with other conditions such as Salmonella sp. or Escherichia coli infection^4^. Class VI, which was added to this classification later, was based on a study of patients with schistosomiasis who were coinfected with hepatitis C virus^30^. In a study conducted in Egypt, cryoglobulins were detected in 8.1% of patients coinfected with *S. mansoni* and hepatitis C virus^31^. To our knowledge, there have been no reports of cases of class II, V, or VI glomerulopathy in Brazil. Although forms of membranous glomerulopathy have been associated with schistosomiasis in the country, those forms are not included in the AFRAN classification^18,19^.

Barsoum et al.^32^, testing the hypothesis that serum levels and deposits of immunoglobulin A (IgA) have a significant role in kidney histology, especially in patients with accompanying liver disease, found IgA deposits in 70% of patients with class III, IV, or V glomerulopathy, compared with 29% in those with class I or II. The authors also found that serum IgA levels were significantly higher in glomerulopathies whose immunofluorescence showed an immune-mediated pattern and that anti-gliadin (IgA) antibody titers were higher in patients with the hepatosplenic form than in those with the intestinal form. That discovery has been supported by the results of an experimental study^33^ and is considered important in the progression of schistosomal nephropathy. However, a study performed in Brazil found that immunofluorescence of kidney biopsies from patients with schistosomal glomerulopathy revealed a higher prevalence of IgM deposition than other immunoglobulins, including IgG^9^.

## Pathogenesis of Glomerular Disease

Although the pathogenesis of schistosomal glomerulopathy remains unclear, it is believed to be multifactorial^10^. Experimental studies have shown that 20% of mice infected with cercariae of *S. mansoni* develop glomerular lesions with deposits of immunoglobulins and complement C3, mainly as mesangial deposition, suggesting a pathogenesis associated with the formation of immune complexes against a schistosome antigen^34^.

The pathogenesis of schistosomal glomerulopathy by immune complexes are associated with a membranoproliferative pattern^9^. The fact that MPGN is often marked by hypocomplementemia, with low levels of complement C3 and C4, suggests that the classical complement pathway is activated^35^. Initially, this histological form and its pathogenesis were associated with hepatosplenic schistosomiasis, in which the presence of portal hypertension and portosystemic shunt would be essential for enhancing the circulation of these immune complexes. In this setting, the liver is bypassed in the clearance of immunoglobulins and schistosome antigens, allowing greater quantities to reach the kidneys^35,36^. However, some studies have documented the presence of the membranoproliferative pattern in the intestinal forms of the disease^9,10^, showing that the hepatosplenic form is not a sine qua non condition for the development of immune complexes and the membranoproliferative pattern^35^.

Schistosomiasis is a disease in which there is chronic antigenemia, and the worm survives in the human host for 15–20 years. Therefore, it is possible that schistosome antigens will passively permeate glomerular structures previously damaged by other agents or areas of normal glomeruli^21,22,23,24^.

**Figure 1:**
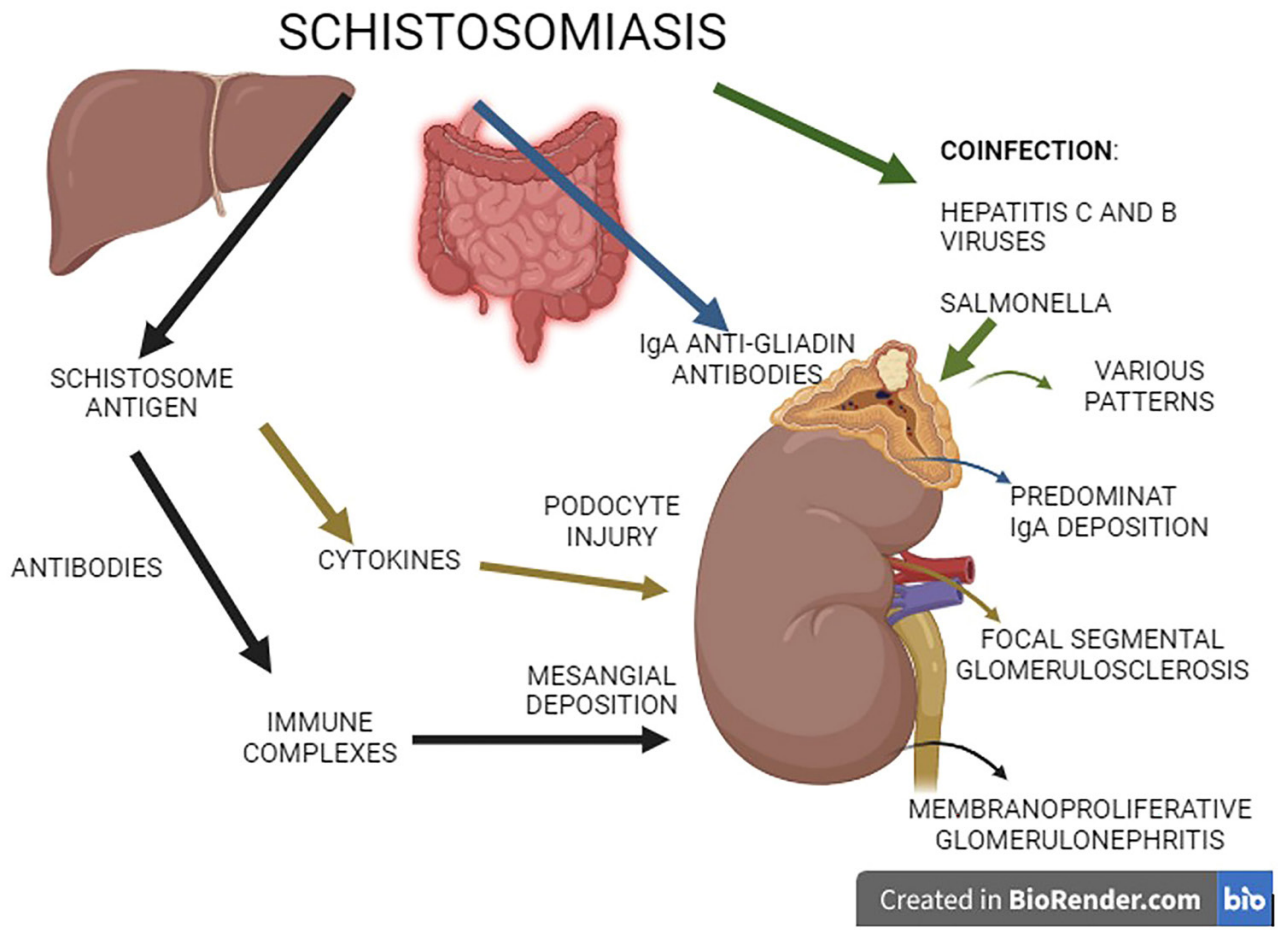
Suggested pathogenesis of glomerulopathy associated with schistosomiasis mansoni.

For other histological patterns, other pathogenic pathways have been proposed. One example is the FSGS pattern, for which it is argued that the diffuse podocyte effacement, characteristic of the form with a nephrotic syndrome presentation, occurs through the direct action of the schistosome antigen on the podocyte, as in HIV, or through the activity of cytokines^35^. Taking these results together, Nussenzveig et al.^35^ suggested that infection with *S. mansoni* causes different pathogeneses for certain patterns of glomerulopathies (Figure 1).

## Diagnosis

The diagnosis of schistosomiasis is based on epidemiological evidence, combined with clinical data, such as the presence of hepatosplenomegaly after exclusion of other causes, such as viral hepatitis. Confirmation requires the detection of parasite eggs in stool or in a rectal biopsy sample, or the identification of serum circulating anti-schistosomiasis antibodies.

### Direct Methods

The first procedures developed for the diagnosis of schistosomiasis were direct diagnostic methods, based on the demonstration of eggs or miracidia in stool samples through parasitological examinations or in tissues through rectal biopsy or biopsy of other organs^28^. Because oviposition (egg laying) in intestinal schistosomes begins approximately 4–6 weeks after infection by cercariae, the available direct egg detection tests are not appropriate for early diagnosis^28^. In 1919, the Brazilian physician Adolpho Lutz devised a series of modifications to the parasitological method of diagnosis (spontaneous sedimentation), described primarily for the identification of *S. mansoni* eggs^37^. These modifications were later refined and standardized in 1934 into the Hoffman-Pons-Janer coprologic method^38^, which is based on the principle of gravity and aims to diagnose intestinal parasites, allowing the concentration of eggs, cysts, oocysts, and larvae of numerous species to be quantified in a fecal sample^38^.

The Kato-Katz thick-smear stool examination, developed in 1972, is still widely used and is the standard method recommended by the WHO^39^. In a stool sample, the level of infection is expressed as eggs per gram (EPG), and the observation of 1 egg on a slide corresponds to the detection of 20–40 EPG, or 5,000–10,000 eggs/day per 250 g of fecal sample. A burden of 1–99 EPG is considered representative of a low-intensity infection^3^. The Kato-Katz method has a high specificity and is simple, requires less work than many other procedures, inexpensive, and easily used in field conditions. However, the method is often not sensitive enough to detect acute, low-intensity infections, such as those seen in travelers or recent immigrants of an area endemic for schistosomiasis or in residents of areas with low prevalence^3^. In addition, the excretion of schistosome eggs varies daily, and excessive dispersion of egg production results in high daily variability in the results of the Kato-Katz test, especially in low-intensity infections or after treatment with praziquantel. The sensitivity of the test can be improved by using multiple stool samples over consecutive days, although that may compromise its simplicity and cost-effectiveness. Compliance with this modified approach is often low, as many individuals are reluctant to provide more than one stool sample^37,38,39^. In suspected cases with negative results on parasitological examinations, rectal biopsy egg counts is employed. This technique is based on the fact that the parasite inhabits the hemorrhoidal plexus where it lays its eggs^27^.

### Indirect Methods

Indirect methods for the diagnosis of schistosomiasis rely on symptoms and clinical signs, as well as on the results of biochemical and immunological analyses^40^. Patient history, such as recent travel to endemic areas or exposure to fresh water for recreational or other activities, can provide an indication of schistosomiasis. Biochemical markers of hepatic fibrosis are currently the focus of research. Serum levels of procollagen peptide (types III and IV), laminin P1 fragment, hyaluronic acid, and fibrosin can be elevated in patients with severe hepatic fibrosis and might decrease after treatment with praziquantel. Ultrasound can be used to visualize pathological changes associated with established active and late chronic schistosomiasis, such as periportal fibrosis and signs of portal hypertension^25^.

Immunological diagnostic approaches include tests that detect schistosome antibodies or circulating schistosome antigens in plasma, serum, urine, or sputum^28^. The most common technique used for antibody detection is enzyme-linked immunosorbent assay (ELISA). Soluble egg antigen, worm antigen, and cationic fraction 6 have high sensitivity but low specificity. ELISA using keyhole limpet hemocyanin as antigen has been shown to be effective in differentiating between acute from chronic schistosomiasis in patients living in endemic areas of Egypt and Brazil^41^. Although these tests can be useful for diagnosing patients from non-endemic areas who visit endemic areas, they are based on antibody titers and have disadvantages, including low specificity, cross-reactivity, and the need to be perform at a referral center, as well as the fact that antibodies can persist after therapy and parasitological cure. Serological tests are useful in field studies to define regions of low endemicity where individual patients have low egg burdens^25^.

### Schistosome Antigen

When schistosomes feed on red blood cells, they regurgitate waste products into the bloodstream. Circulating anodic antigens (CAAs) and circulating cathodic antigens (CCAs), which are proteoglycans bearing negative and positive charges, respectively, are a reliable marker of active Schistosoma infection, indicating the presence of live worms within the host. These antigens can be detected early, even before the parasites begin egg production^3^. Renal biopsies from individuals with active *S. mansoni* infection have shown deposition of CAAs and CCAs^4^.

### New Renal Biomarkers

Infection with *S. mansoni* can induce a chronic kidney inflammatory state, evidenced by an increase in urinary CCL2, chemokine expressed at sites of injury and inflammation, that is not resolved by specific treatment of the offending agent^42^. In a study conducted in the Brazilian state of Ceará, Hanemann et al.^43^ demonstrated that patients with schistosomiasis mansoni had high levels of urinary CCL2 that correlated positively with albuminuria.

Vascular endothelial growth factor (VEGF), which is synthesized by podocytes and acting in the endothelium pathway, has been studied for its correlation with CAA^44^. A recent study in a population living in an endemic area showed that those who tested positive for *S. mansoni*, even without clinically evident kidney disease, had significantly higher urinary levels of VEGF than those who tested negative for schistosomiasis^42^.

The 2021 Kidney Disease Improving Global Outcomes (KDIGO) guidelines^45^ recommend testing for appropriate endemic coinfection (Salmonella sp., hepatitis B virus, hepatitis C virus, or HIV) because targeted treatment can reduce the aggressiveness of an underlying glomerulonephritis or schistosomiasis sequela.

## Treatment

Reducing the morbidity and mortality of schistosomiasis requires early detection and prompt treatment of all carriers, to prevent the cumulative pathogenic activity of *S. mansoni* eggs, which can affect certain organs, especially the liver, resulting in portal hypertension and other severe forms of schistosomiasis.

Praziquantel is the first-line treatment for all types of schistosomiasis. Since its discovery in the mid-1970s, its safety and efficacy have ensured its widespread use^25^. The treatment with praziquantel aims to control the production of schistosomiasis eggs by eliminating adult worms, so that the resulting morbidity, including associated complications and mortality, are minimized. However, the treatment does not reverse the complications associated with tissue fibrosis. Because praziquantel is not effective against the early stages of immature schistosomes, affecting exclusively adult worms, other treatment alternatives should be considered in early disease^25^. Another means of reducing the geographic spread of schistosomiasis is through early detection and treatment of carriers of the disease. One of the difficulties in the early detection of *S. mansoni* carriers is that the infection can evolve silently until the onset of a severe form.

Human and animal studies have shown that curing schistosomiasis does not halt the progression of glomerulopathy. This suggests that, despite the importance of schistosome antigens in the initiation of glomerular lesions, other factors, such as autoimmunity, antinuclear antibodies, rheumatoid factor, and cryoglobulins, play a role in the subsequent progression of the lesions^10^.

It remains uncertain whether immunosuppressive therapy is effective in the treatment of schistosomal glomerulopathy. Martinelli et al.^46^ stated that there is no specific treatment for schistosome-related glomerular lesions. The authors showed that lesions categorized as AFRAN class I regress with treatment of the infection, and that AFRAN class II lesions resolve with antischistosomal treatment (e.g., praziquantel) and anti-salmonella therapy with the combination of cotrimoxazole (960 mg, twice daily) and ampicillin (1 g every 6 hours) or with a quinolone, such as ciprofloxacin (1 g, twice daily). They also demonstrated that lesions categorized as AFRAN class III through V do not respond to infection eradication, which may require corticosteroids or immunosuppressive agents.

In another study, Martinelli et al.^47^ evaluated the response to immunosuppressive therapy in patients with FSGS and the hepatosplenic form of schistosomiasis mansoni. Of the 15 patients evaluated, 10 received immunosuppressive therapy. Seven were classified as resistant to therapy, although one of those patients became responsive to steroids during follow-up. All three patients who responded to steroids received concurrent treatment with cyclophosphamide to prevent frequent relapses. Immunosuppressive therapy was well tolerated, without related complications.

Currently, the role of immunosuppressive therapy in patient remission is unclear. The 2021 KDIGO guidelines^45^ recommend that patients with schistosomal infection and glomerulopathy be treated with an appropriate antiparasitic agent at a dosage and duration sufficient to eradicate the microorganism. Current guidelines provide no specific indication for the use of immunosuppressive agents. The uncertainty surrounding their role in schistosomiasis-associated glomerulopathy is closely related to the unclear pathogenesis, especially of MPGN and FSGS. However, the progression to chronic kidney disease that occurred in 64% of our patients with schistosomiasis-associated glomerulopathy over an average follow-up period of 5 years^9^ makes us believe that there is a need to better investigate the pathogenetic pathways of this disease, propose adequate therapy based on the pathogenesis, and better evaluate the evolution of kidney disease.

## Conclusion

In conclusion, the pathogenesis of glomerular involvement by schistosomiasis mansoni is not completely clear, and therefore, whether antiparasitic treatment alone will be sufficient to improve kidney survival is uncertain. It is important to note that many patients with schistosomiasis-associated glomerulopathy are likely to evolve to chronic kidney disease. The high risk of an unfavorable renal outcome together with the high prevalence of this tropical disease, represents a major challenge for improving public health.

## Data Availability

The data used in this study are not publicly available.
