# Supplementary material for: Glomerulopathy associated with schistosomiasis mansoni
Source: J Bras Nefrol. 2025 Oct 6;48(1):e20250084. doi: 10.1590/2175-8239-JBN-2025-0084en (PMC12534032; doi:10.1590/2175-8239-JBN-2025-0084en)
Supplement: Translation

# Translation: Glomerulopatia associada à esquistossomose mansônica

**DOI:** 10.1590/2175-8239-JBN-2025-0084pt

**Authors:** Irma Bandeira de Sousa Pontes, Amaro Nunes Duarte, Cristiane Bitencourt Dias

**Affiliations:** 1 Universidade de São Paulo, Faculdade de Medicina, Hospital das Clínicas, Laboratório de Fisiopatologia Renal, São Paulo, SP, Brasil.; 2 Universidade de São Paulo, Faculdade de Medicina, Departamento de Patologia, São Paulo, SP, Brasil.

**Keywords:** Esquistossomose, Glomerulonefrite Membranoproliferativa, Imunossupressores, Biomarcadores, Antígenos

## Abstract

A esquistossomose é uma doença parasitária, diretamente relacionada às condições de saneamento precário. No Brasil, 42 milhões de pessoas vivem em áreas endêmicas para essa doença, que ainda é considerada um grave problema de saúde pública. O rim é um dos órgãos tipicamente afetados pelo *Schistosoma mansoni*, com descrições de alterações tubulares, lesão renal aguda e doença glomerular. A incidência de doença glomerular é de 5 a 6% em pacientes com esquistossomose ativa, enquanto aumenta para até 15% na forma hepatoesplênica, sendo o padrão histológico mais descrito a glomerulonefrite membranoproliferativa. Embora a patogênese da doença glomerular associada à esquistossomose seja incerta, o envolvimento de imunocomplexos, especialmente na glomerulonefrite membranoproliferativa, formados contra um antígeno esquistossômico depositado nos glomérulos, é considerado. A incerteza da patogênese faz com que o tratamento seja um desafio, pois somente o uso de medicação antiparasitária não impede a progressão para doença renal crônica, levantando questionamentos sobre a necessidade do uso de medicações imunossupressoras. Assim, o objetivo desta revisão foi descrever as informações ditas clássicas sobre a doença glomerular associada à esquistossomose e explorar seus prováveis mecanismos patogênicos, a fim de promover discussões futuras sobre o desenvolvimento de melhores opções de tratamento.

## Introdução

A esquistossomose é uma doença parasitária causada por helminto pertencente à classe *Trematoda*. Foi identificada pela primeira vez em 1852, no Cairo, pelo alemão Theodor Bilharz, durante necropsia de veias mesentéricas. Por esse motivo, a denominação de “bilharziose” ou “bilharzíase” é utilizada como sinônimo para esquistossomose^1^. Posteriormente, o pesquisador brasileiro Manoel Augusto Pirajá da Silva descobriu e identificou o *Schistosoma mansoni*, agente etiológico da esquistossomose mansoni, em 1908, no estado da Bahia^2^.

As espécies causadoras dessa doença em seres humanos são o *Schistosoma haematobium*, encontrado principalmente na África; o *Schistosoma japonicum*, endêmico da China e do Extremo Oriente; e o *Schistosoma mansoni*, na América Latina. O *Schistosoma guineensis*, o *Schistosoma intercalatum* e o *Schistosoma mekongi* também infectam o ser humano, porém apresentam menor prevalência global^3^.

A transmissão desse parasita ocorre por meio da penetração, na pele e nas mucosas, das cercárias - forma do parasita liberada em água doce por caramujos que ali vivem e em locais onde pessoas têm acesso para banhos e mergulhos. A perpetuação da transmissão da doença depende da presença do homem infectado, que excreta ovos do helminto pelas fezes, e dos caramujos aquáticos, que atuam como hospedeiros intermediários, liberando larvas infectantes do verme nas coleções hídricas utilizadas pelos seres humanos. Após a penetração no homem, os órgãos tipicamente afetados incluem os rins, o trato urinário, o intestino e o fígado^4^.

O envolvimento renal na esquistossomose é variável. A incidência de doença glomerular é de 5 a 6% em pacientes com esquistossomose ativa, enquanto pode aumentar até 15% na forma hepatoesplênica^5^. O tipo histológico relatado com maior frequência é a glomerulonefrite membranoproliferativa (GNMP)^6–8^. A glomerulopatia associada à esquistossomose é estudada há mais de meio século, com diversos trabalhos confirmando essa associação, cuja patogênese apresenta componente multifatorial, porém ainda não totalmente esclarecida, o que torna seu tratamento um desafio^9^. A cura da esquistossomose por meio do tratamento antiparasitário não interrompe a progressão da doença glomerular. Esses dados podem sugerir que, apesar da importância dos antígenos relacionados ao verme no início da lesão glomerular, outros fatores, como autoimunidade e produção de citoquinas, podem desempenhar um papel de agravamento e contribuir para pior evolução desse acometimento glomerular^9,10^.

Outros problemas renais também ocorrem, como a lesão renal aguda descrita por Duarte et al.^11^ em 43,3% dos pacientes internados com a forma hepatoesplênica da esquistossomose, bem como a disfunção tubular caracterizada por déficit de concentração urinária e déficit de acidificação urinária, demonstrada pelos mesmos autores em outro estudo com pacientes com a forma hepatoesplênica compensada^12^. Essas últimas formas de acometimento renal descritas não serão objeto deste artigo. Assim, o objetivo desta revisão foi descrever as informações ditas clássicas sobre a doença glomerular associada à esquistossomose e explorar seus prováveis mecanismos patogênicos, a fim de promover discussões futuras sobre o desenvolvimento de melhores opções de tratamento.

## Epidemiologia da Doença Glomerular Associada à Esquistossomose

Apesar de ser uma das doenças infecciosas tropicais mais prevalentes, a esquistossomose é agrupada na categoria denominada Doenças Tropicais Negligenciadas^13^. De acordo com a Organização Mundial da Saúde (OMS), em 2018, a esquistossomose ocupava o segundo lugar, depois da malária, na escala de doenças infecciosas tropicais, em razão de sua importância e repercussão socioeconômica. Afeta quase 240 milhões de pessoas em todo o mundo, sendo que mais de 700 milhões vivem em áreas endêmicas. Entre as parasitoses que afetam o homem, é uma das mais disseminadas globalmente^14^.Na Região das Américas, um total de 10 países e territórios são considerados endêmicos, e 25 milhões de pessoas estão em risco^15^.

No Brasil, 42 milhões de pessoas vivem em áreas endêmicas para essa doença, que ainda é considerada um grave problema de saúde pública nacional, sendo as regiões Norte e Nordeste do país as que apresentam mais notificações de casos, mesmo após a implementação de programas de controle da esquistossomose^16,17^. O rim é um dos órgãos tipicamente afetados pelo *S. mansoni*, destacando-se aqui as doenças glomerulares associadas a esse verme. Alguns padrões histológicos foram descritos, mas os mais frequentes são a GNMP, a glomeruloesclerose segmentar focal (GESF), a glomerulonefrite mesangial proliferativa e a nefropatia membranosa^9,18^.

O padrão histológico de doença glomerular mais associado à esquistossomose irá depender da forma clínica de apresentação da doença. As formas hepatoesplênicas estão mais associadas à GNMP. Esse dado pode ser melhor observado em dois trabalhos realizados em nosso serviço e descritos na Tabela 1, onde, nas formas intestinais, a prevalência de GNMP é de 20,8% e, na forma hepatoesplênica, de 70,8%. Ocorre o predomínio da glomerulonefrite proliferativa mesangial na forma intestinal, e o padrão de GESF é observado em 25% em ambas as formas de esquistossomose^9,19^. A ideia de nefropatia membranosa associada à esquistossomose não é bem aceita, principalmente porque não está incluída na classificação da Associação Africana de Nefrologia (AFRAN). No entanto, dois estudos realizados no Brasil descreveram esse padrão glomerular em um total de 14 casos de esquistossomose, se considerarmos os dois estudos^18,19^.

**Tabela 1:** Comparação das prevalências dos padrões de doença glomerular pela forma de apresentação clínica da esquistossomose

Dados à época da biópsia renal	Abensur et al.^19^	Neves et al.^9^
Número de pacientes	24	24
Idade (anos)	29,9 ± 12,6	38,58 ± 9,83
Sexo masculino (%)	58,3	79,1
Creatinina sérica (mg/dl)	1,40 ± 0,7	1,58 ± 0,80
Hipertensão arterial sistêmica (%)	45,8	75
Síndrome Nefrótica (%)	100	33,3
Forma hepatoesplênica (%)	0	36,4
Proliferativa Mesangial (%)	33,3	4,1
Glomeruloesclerose segmentar e focal (%)	25	25
Glomerulonefrite membranoproliferativa (%)	20,8	70,8
Nefropatia Membranosa (%)	8,3	0
Doença de Lesão Mínima (%)	8,3	0
Outras (%)	4,2	0

Já se passaram nove anos desde que a OMS publicou seu primeiro roteiro voltado para doenças tropicais negligenciadas, com metas definidas para 2020. Embora tenha havido progressos notáveis, várias dessas metas permanecem não alcançadas. Em resposta, um novo roteiro foi desenvolvido após uma consulta global abrangente, delineando estratégias para o período de 2021 a 2030*.* Este identifica as lacunas críticas e as ações necessárias para atingir as metas de 2030 e estabelece metas e marcos globais para prevenir, controlar, eliminar ou erradicar 20 doenças e grupos de doenças, incluindo a esquistossomose^20^.

## Ciclo de Vida do Schistosoma Mansoni

Os esquistossomos são vermes sanguíneos que parasitam o sistema venoso de uma ampla variedade de animais, incluindo aves, bovinos e primatas^4^. O ciclo de vida dos principais esquistossomos é bastante semelhante; as diferenças consistem principalmente nas espécies intermediárias de caracóis envolvidas e na distribuição dos tecidos dentro dos hospedeiros definitivos^21^. O ciclo de vida do parasito é constituído por duas fases: a assexuada, que se inicia com a penetração de miracídios no hospedeiro intermediário e culmina com a eliminação das cercárias, e a fase sexuada, que ocorre no hospedeiro definitivo^22^. Dois hospedeiros estão envolvidos: o homem (hospedeiro definitivo) e os caramujos de água doce do gênero *Biomphalaria* (hospedeiro intermediário)^21^.

O homem contaminado excreta, pelas fezes, ovos do helminto que podem contaminar o ambiente aquático. Os ovos eclodem e liberam larvas ciliadas (miracídios) nas coleções hídricas utilizadas pelos seres humanos. Estas penetram nos caramujos, onde sofrem mudanças estruturais, passando de esporocisto primário a esporocisto secundário e originando novas larvas (cercárias), que abandonam o caramujo e permanecem na água até penetrarem na pele do homem^13,17^. A maior liberação ocorre em momentos em que o calor e a luz solar são mais intensos (entre 10h e 16h). Esse momento coincide com o período em que uma maior quantidade de pessoas está em contato com a água para a prática do banho ou para recreação aquática^2^. As larvas bifurcadas (cercárias) penetram na pele e nas mucosas, perdem a cauda no processo, transformam-se em esquistossômulos e entram na circulação sanguínea por meio dos capilares e vasos linfáticos. Elas são então transportadas por todo o corpo pelo fluxo sanguíneo por vários dias antes de ficarem presas na veia porta^2^.

O *S. mansoni* é habitualmente hóspede das vênulas tributárias do sistema porta, particularmente das veias mesentéricas superiores e inferiores, do plexo hemorroidário e da porção intra-hepática da veia porta. Geralmente, esses vermes realizam migrações dentro do mesmo vaso ou de um para outro por meio de anastomoses^13^. No fígado, as formas jovens se alimentam, crescem e diferenciam-se sexualmente. Elas migram, então, para o intestino, no qual adquirem a forma adulta. É nesse local que os vermes adultos se acasalam. Após o acasalamento, ocorre a liberação de ovos, que se dirigem para a luz intestinal e são eliminados com as fezes. Em 27 dias após a penetração na pele, podem se transformar em vermes adultos. A postura de ovos pode começar após o 30º dia e, a partir do 40º dia, podem ser encontrados ovos nas fezes. A presença de ovos nas fezes humanas ocorre, em média, por cinco anos, embora existam relatos de pacientes eliminando ovos até 30 anos após deixarem a área endêmica^23^.

## Quadro Clínico

### Manifestações Imediatas

Na pele, local da penetração pelas cercárias, a resposta imune inata às larvas moribundas ou mortas dá origem a reações de hipersensibilidade^3^ e pode causar uma reação inflamatória pruriginosa maculopapular, que varia em tamanho de 1 a 3 cm, chamada dermatite cercariana^24,25^. Durante a passagem pela epiderme e derme, ocorre reação de hipersensibilidade do tipo imediata, com ativação de vários componentes da resposta imune inata. Em dois dias, organiza-se um infiltrado de polimorfonucleares, mononucleares e células de *Langerhans*, além de produção local de proteína inflamatória de macrófago (CCL3/ MIP-1α) e interleucinas (IL), como a IL1b, IL6, IL12p40 e IL10. Depois de quatro a cinco dias, este cenário ainda é predominante, podendo-se observar o influxo de linfócitos T CD4+ e a produção de IL-12p40, interferon-gama (IFN-γ) e IL-4, os quais se reduzem na segunda semana^26^. A dermatite cercariana normalmente ocorre em indivíduos expostos ao *Schistosoma* pela primeira vez, como viajantes e migrantes para áreas endêmicas de esquistossomose^3^.

### Esquistossomose Aguda

Após a penetração cercariana bem-sucedida e formação do esquistossômulo, a infecção pode prosseguir para um estágio agudo, também conhecido como febre de Katayama, em homenagem a um distrito de Hiroshima, no Japão, onde *S. japonicum* foi detectado pela primeira vez em um ser humano^3^. É frequente uma história de contato com água contaminada de 14 a 84 dias antes da apresentação^25^.

Os sintomas são causados por reações de hipersensibilidade sistêmica e pela formação de imunocomplexos em resposta a antígenos liberados durante a migração do esquistossômulo ou no início da deposição de ovos^3^. Incluem febre, cefaleia, mialgias generalizadas, dor no quadrante superior direito e diarreia com sangue. Sintomas respiratórios foram relatados em até 70% das pessoas infectadas com o *S. Mansoni*, e pode haver evidência radiológica de pneumonite intersticial. A hepatomegalia dolorosa geralmente está presente, e a esplenomegalia ocorre em um terço dos casos. A meningite asséptica é rara. Nessa fase, todos os pacientes apresentam eosinofilia, e a maioria tem testes sorológicos positivos para esquistossomose^25^.

A esquistossomose aguda raramente é observada em pessoas que vivem em áreas endêmicas^27^. Essa falta de suscetibilidade a sintomas agudos pode ser devida à dessensibilização intraútero, resultando em menor resposta imunológica aos antígenos do esquistossomo em bebês nascidos de mães infectadas, ou possivelmente à exposição repetida às cercárias, que induzem a produção de IL-10 por células T CD4+ na pele, resultando em uma resposta imune regulatória^3^.

## Esquistossomose Crônica

A esquistossomose crônica deve-se, principalmente, à reação inflamatória granulomatosa contra os ovos de esquistossomo depositados em diferentes órgãos e tecidos^28^. Esses ovos secretam ativamente glicoproteínas antigênicas, que têm a função de facilitar a passagem destes através dos vasos sanguíneos, onde são depositados^3^. A intensidade e a duração da infecção determinam a quantidade de antígeno liberado e a gravidade de doença fibro-obstrutiva crônica^25^. Alguns ovos permanecem nos capilares da mucosa do intestino, ao passo que outros são carreados pela circulação mesentérica até o fígado, onde “encalham” nos sinusoides hepáticos. A liberação de antígenos solúveis a partir dos ovos induz a mobilização de macrófagos, eosinófilos, linfócitos e plasmócitos, havendo, nesse processo, a mediação por fator de necrose tumoral α (TNFα), linfócitos T CD4+, Th1, Th2 e CD8+. Os macrófagos entram em contato com o ovo, formando massas sinciciais multinucleadas; algumas dessas células transformam-se em fibroblastos, orientando a organização de camadas concêntricas em toda a espessura do granuloma, com ampla produção de colágeno^26^. Os granulomas periovulares são grandes, com características predominantemente necrótico-exsudativas, e aparecem como grânulos translúcidos disseminados na superfície serosa do fígado e dos intestinos^24^. As altas respostas proliferativas de linfócitos, induzidas por antígenos de ovo solúveis nesta fase da infecção, diminuem progressivamente à medida que a infecção se torna crônica^3^.

A fase crônica da doença pode apresentar-se de maneira polimórfica. Compreende as formas intestinal, hepatointestinal e hepatoesplênica, os distúrbios vasculares pulmonares, as formas pseudoneoplásicas, a nefropatia esquistossomótica e as lesões ectópicas^26^. A forma hepatointestinal nada mais é do que a forma intestinal associada à hepatomegalia. A observação da forma intestinal pura é incomum, podendo ser assintomática ou acompanhada de dor abdominal intermitente, desconforto, perda de apetite e, às vezes, diarreia com sangue, em que o paciente apresenta baixa carga de vermes^3,28^. Na apresentação hepatoesplênica, há formação de granuloma causado pela deposição de ovos no fígado, resultando em fibrose periportal^28^. A fibrose periportal é geralmente observada em adultos e, ocasionalmente, em adolescentes que vivem em áreas com transmissão muito alta da esquistossomose. A gravidade da fibrose periportal correlaciona-se, em parte, com a intensidade e a duração da infecção. A oclusão do ramo portal, resultante de fibrose periportal, pode levar à hipertensão portal acentuada, frequentemente acompanhada por aumento grave e endurecimento do baço. Em alguns casos, a hipertensão portal leva ao desenvolvimento de varizes esofágicas, que podem se romper, com alto risco de hematêmese fatal. Outras complicações incluem ascite, atraso no crescimento, atraso puberal e anemia grave. Contudo, em contraste com a cirrose hepática de etiologia alcoólica ou de outras origens, na esquistossomose, mesmo na presença de fibrose periportal acentuada e hipertensão portal, não se observa dano aos hepatócitos, e as enzimas hepáticas permanecem dentro dos valores de normalidade^3^. A neuroesquistossomose pode ser um dos desfechos clínicos mais graves da doença, sendo causada pela resposta inflamatória ao redor dos ovos depositados no plexo venoso cerebral ou espinhal.

A esquistossomose pulmonar é causada por desvio portocava, no qual o sangue venoso contorna o fígado por meio de veias colaterais que conectam a veia porta à veia cava superior. Nessa situação, os ovos do parasita são transportados até os capilares pulmonares, onde induzem a formação de granulomas na área perialveolar. Esses granulomas podem levar à fibrose e resultar em hipertensão pulmonar e cor pulmonale^3^.

Pacientes com acometimento glomerular podem ser assintomáticos, fato que contribui para a complexidade em estimar a real incidência desse acometimento, incluindo o fato de que o envolvimento glomerular também pode ser autolimitado^29^. Indivíduos com esquistossomose podem apresentar-se com síndrome nefrótica, proteinúria subnefrótica isolada, glomerulonefrite aguda, síndrome mista ou doença renal crônica^9,19^.

Na glomerulopatia esquistossomótica, o mecanismo fisiopatogênico difere das demais formas clínicas da esquistossomose, nas quais a formação de granulomas ao redor dos ovos representa o evento mais importante. Nesse contexto, a reação antígeno-anticorpo, mediada pela formação de imunocomplexos depositados nos glomérulos, representa o provável mecanismo patogênico desse acometimento renal^26^. Os mecanismos patogênicos da doença glomerular serão abordados em tópico específico adiante.

## Padrões Histológicos Glomerulares

Os tipos histológicos mais frequentemente relatados são GNMP e GESF, geralmente associadas às apresentações clínicas de síndrome mista e síndrome nefrótica, respectivamente^9,19^. A diversidade das lesões histológicas renais já encontradas motivou uma classificação clínico-patológica da glomerulopatia esquistossomótica, endossada em 1992 pela AFRAN, que reconheceu seis categorias de doenças glomerulares (Quadro 1)^4,5^.

**Quadro 1:** 1 Classificação histológica segundo a afran

Classe	Histologia renal	Apresentação clínico-laboratorial
I	Glomerulonefrite proliferativa mesangial	Micro-hematúria e proteinúria subnefrótica
II	Glomerulonefrite exsudativa difusa	Síndrome nefrítica aguda
III	Glomerulonefrite membranoproliferativa	Síndrome mista e hipertensão arterial
IV	Glomeruloesclerose segmentar e focal	Síndrome nefrótica e hipertensão arterial
V	Amiloidose	Síndrome nefrótica
VI	Glomerulonefrite crioglobulinêmica	Púrpura, vasculite, artrite, hipertensão arterial e síndrome mista

Nessa classificação, a classe II ocorreria quando em associação com salmonelose e clinicamente se assemelharia às formas de glomerulonefrite difusa aguda pós-estafilocócicas/estreptocócicas, incluindo o consumo apenas do complemento C3. Na classe V, a amiloidose seria por depósito de proteína amiloide A e também ocorreria em associação a outras infecções, como salmonelose e *E coli* ^4^. A classe VI foi adicionada posteriormente a essa classificação, a partir de estudo com pacientes com vírus C da hepatite e esquistossomose^30^. Em estudo no Egito, a detecção de crioglobulinas ocorreu em 8,1% dos pacientes coinfectados^31^. No Brasil, as classes II, V e VI não foram descritas; contudo, formas de glomerulopatia membranosa foram associadas à esquistossomose neste país, mas não são contempladas na classificação da AFRAN^18,19^.

Barsoum et al.^32^, tentando avaliar uma provável maior importância de níveis séricos e depósitos na histologia renal de imunoglobulina A (IgA), em especial nos pacientes com doença hepática associada, encontraram depósito de IgA em 70% das classes III, IV e V, versus 29% nas classes I e II. Além disso, demonstraram aumento significativo de IgA sérica nas glomerulopatias cuja imunofluorescência mostrava um padrão imunomediado e aumento do anticorpo anti-gliadina (IgA) nos pacientes com a forma hepatoesplênica da doença, versus a forma intestinal. Esta descoberta tem suporte experimental^33^ e foi considerada importante na progressão da nefropatia esquistossomótica. Contudo, um estudo realizado no Brasil demonstrou que a imunofluorescência de biópsias renais com glomerulopatia associada à esquistossomose demonstrava uma prevalência maior de IgM em relação às demais imunoglobulinas, inclusive à IgG^9^.

## Patogênese da Doença Glomerular

A patogênese da doença glomerular associada à esquistossomose não é bem conhecida; entretanto, acredita-se que tenha um componente multifatorial^10^. Trabalhos experimentais com camundongos infectados com cercárias de *S mansoni* desenvolveram lesões glomerulares com depósitos de imunoglobulinas e complemento C3, principalmente com deposição mesangial, sugerindo uma patogênese associada à formação de imunocomplexos contra um antígeno esquistossômico encontrado em 20% desses animais^34^.

Essa patogênese por imunocomplexos estaria associada ao padrão membranoproliferativo^9^. A GNMP é frequentemente marcada por hipocomplementemia, com níveis baixos de complemento C3 e C4, sugerindo ativação da via clássica do complemento^35^. Inicialmente, essa forma histológica e sua patogênese foram associadas à esquistossomose hepatoesplênica, onde a presença de hipertensão porta e *shunt* portosistêmico seriam essenciais na potencialização da circulação desses imunocomplexos, associado ao fato de o fígado ser excluído do processo de clareamento de imunoglobulinas e dos antígenos esquistossomóticos, que são ofertados aos rins em maior quantidade^35,36^. Todavia, alguns estudos já documentaram a presença do padrão membranoproliferativo nas formas intestinais da doença^9,10^, mostrando que a forma hepatoesplênica não é condição *sine qua non* para o desenvolvimento de imunocomplexos e do padrão membranoproliferativo^35^.

A esquistossomose é uma doença com antigenemia crônica, em que o verme sobrevive no hospedeiro humano por 15–20 anos. Portanto, é possível que os antígenos esquistossômicos possam impregnar passivamente (a chamada armadilha não imunológica) em estruturas glomerulares anteriormente lesionadas por outros agentes ou impregnar em área glomerular normal. Se assim for, glomerulopatias que, embora já tenham sido descritas em conjunto com doenças infecciosas^21–24^, podem ser primárias, pelo depósito passivo de antígeno no glomérulo.

Para outros padrões histológicos, outras vias patogênicas são defendidas. Um exemplo seria o padrão de GESF, onde se defende que o desabamento difuso dos podócitos, característica da forma com apresentação de síndrome nefrótica, ocorreria pela ação direta do antígeno esquistossômico no podócito, a exemplo do que ocorre no HIV, ou pela ação de citoquinas^35^. Tendo em conjunto estes resultados, Nussenzveig et al. sugerem que a infecção por *S. mansoni* promove diferentes patogêneses para determinados padrões de glomerulopatias (Figura 1)^35^.

**Figura 1:** Patogênese sugerida da glomerulopatia associada à esquistossomose mansônica.

## Diagnóstico

O diagnóstico de esquistossomose é baseado em evidências epidemiológicas, combinado a dados clínicos, como a presença de hepatoesplenomegalia, descartadas outras etiologias, como as hepatites virais. Associado a isso, é necessária a pesquisa de ovos do verme nas fezes, a realização de biópsia retal ou a detecção sérica de anticorpos da esquistossomose.

### Métodos Diretos

Os métodos de diagnóstico direto foram os primeiros procedimentos diagnósticos desenvolvidos e baseiam-se na demonstração de ovos ou de miracídios em amostras de fezes, por meio dos exames parasitológicos, ou em tecidos, pela biópsia retal ou de outros órgãos^28^. Como a oviposição (postura de ovos) nos esquistossomos intestinais começa cerca de 4 a 6 semanas após a infecção pela cercária, nenhum dos testes de detecção direta de ovos disponíveis é apropriado para o diagnóstico precoce^28^. Em 1919, o médico brasileiro Adolpho Lutz desenvolveu uma série de modificações no método parasitológico (sedimentação espontânea) de diagnóstico, descrito primordialmente para a detecção de ovos de *Schistosoma mansoni* ^37^. Posteriormente, foi mais bem embasada por Hoffman, Pons & Janer no ano de 1934. Consiste em uma técnica respaldada na lei gravitacional, que objetiva diagnosticar parasitos intestinais, permitindo a concentração de ovos, cistos, oocistos e larvas de inúmeras espécies por meio de uma amostra fecal^38^.

A técnica de esfregaço de fezes espessas, baseada na microscopia Kato-Katz, desenvolvida em 1972, ainda é amplamente utilizada e é o método padrão recomendado pela OMS^39^. O nível de infecção é expresso por ovos por grama de fezes (OPG), e a observação de 1 ovo em uma lâmina corresponde à detecção de 20–40 OPG, ou 5.000–10.000 ovos por porção fecal diurna de 250g. Valores de 1–99 OPG são considerados infecções de baixa intensidade^3^. Esse método tem um alto nível de especificidade, é simples, menos trabalhoso que muitos outros procedimentos, é barato e pode ser facilmente utilizado em condições de campo. Contudo, este método muitas vezes não é suficientemente sensível para detectar infecções agudas de baixa intensidade, como as de viajantes que regressam ou de imigrantes recentes de uma área endêmica de esquistossomose, ou em áreas com baixa prevalência^3^. Além disso, há variação diária na excreção de ovos de esquistossomo, e a dispersão excessiva da produção de ovos resulta em alta variabilidade diária nos resultados dos testes de Kato-Katz, especialmente para infecções de baixa intensidade ou após tratamento com prazinquatel. A sensibilidade do teste pode ser melhorada utilizando múltiplas amostras de fezes durante vários dias consecutivos, mas isso pode comprometer a sua simplicidade e relação custo-eficácia. Esta abordagem modificada pode sofrer de baixa adesão devido à relutância dos indivíduos em fornecer mais de uma amostra de fezes^37–39^. A pesquisa de ovos pela biópsia retal é utilizada em casos suspeitos com exames parasitológicos negativos. A ideia dessa pesquisa se deve ao fato de o parasita habitar o plexo hemorroidário e aí fazer a postura dos ovos^27^.

## Métodos Indiretos

Os métodos indiretos para o diagnóstico dependem de sintomas, sinais clínicos e de análises bioquímicas e imunológicas^40^. Nesse caso, informações de anamnese, como viagens recentes a áreas endêmicas e exposição à água doce por meio de atividades recreativas ou outras, são importantes e podem fornecer uma indicação de esquistossomose. Marcadores bioquímicos de fibrose hepática são atualmente foco de pesquisas. Os níveis séricos de peptídeo procolágeno (tipos III e IV), fragmento P1 da laminina, ácido hialurônico e fibrosina podem estar elevados em pacientes com fibrose hepática grave e podem diminuir após o tratamento com praziquantel. A ultrassonografia pode ser usada para visualizar alterações patológicas associadas à esquistossomose crônica ativa e tardia estabelecida, como fibrose periportal e sinais de hipertensão portal^25^.

O diagnóstico imunológico inclui testes que detectam anticorpos antiesquistossômicos ou antígenos esquistossômicos circulantes no plasma, soro, urina ou escarro^28^. A técnica mais comum usada na detecção de anticorpos é o ensaio imunoenzimático (ELISA). O antígeno solúvel do ovo, o antígeno do verme e a fração catiônica 6 apresentam alta sensibilidade, mas baixa especificidade. Um teste ELISA usando hemocianina de lapa como antígeno demonstrou ser eficiente ao diferenciar a esquistossomose aguda da crônica em pacientes que vivem em áreas endêmicas do Egito e do Brasil^41^.Embora esses testes possam ser úteis para diagnosticar pacientes de áreas não endêmicas que visitam áreas endêmicas, eles são baseados em anticorpos, sofrem de baixa especificidade, persistência após quimioterapia, reatividade cruzada, necessidade de centro de referência para realizá-los e persistência após a cura parasitológica. Os testes sorológicos são úteis em estudos de campo para definir regiões de baixo nível de endemicidade, onde pacientes individuais apresentam baixas cargas de ovos^25^.

### Antígeno Circulante do Gênero *Schistosoma*

Quando os esquistossomos se alimentam de glóbulos vermelhos, eles regurgitam resíduos na corrente sanguínea. A detecção de antígenos circulantes anódicos (CAAs) e catódicos (CCAs), que são proteoglicanos com cargas negativa e positiva, respectivamente, representa um marcador confiável de infecção ativa por esquistossomos, indicando a presença de vermes vivos no hospedeiro. Esses antígenos podem ser identificados precocemente, antes mesmo da oviposição pelos parasitas.^3^ Biópsias renais de indivíduos com *S. mansoni* ativo mostraram depósitos de antígenos circulantes (CAA e CCA)^4^.

### Novos Biomarcadores Renais

Diversos biomarcadores têm sido testados para diagnosticar ou prever glomerulopatias associadas à esquistossomose. A Proteína Quimiotática de Monócitos-1 (MCP-1/CCL2) é uma proteína expressa em locais de lesão e inflamação, direcionada ao recrutamento de macrófagos, que se ligam ao receptor de quimiocina para promover a adesão e quimiotaxia de macrófagos^42^. Hanemann et al., 2013, em estudo realizado no estado do Ceará/Brasil, demonstraram que pacientes com esquistossomose mansoni apresentavam níveis elevados de MCP-1 urinária (CCL2), que se correlacionavam positivamente com albuminúria^43^.

O Fator de Crescimento Endotelial Vascular (VEGF), que é sintetizado pelos podócitos e tem sua via efetiva no endotélio, foi estudado para uma provável correlação com a CAA^44^. Um estudo recente, em uma população que vive em uma área endêmica, mostrou que aqueles que testaram positivo para *S. mansoni*, mesmo sem doença renal clínica, apresentavam níveis urinários de VEGF significativamente mais elevados em relação ao grupo testado negativo para esquistossomose^42^.

As diretrizes do KDIGO de 2021^45^ orientam a realização de testes para coinfecções endêmicas apropriadas (Salmonella, HBV, HCV, HIV), pois o tratamento direcionado pode alterar a agressividade de uma glomerulonefrite subjacente ou as sequelas da esquistossomose.

## Tratamento

A redução da morbimortalidade da esquistossomose requer a detecção precoce e o pronto tratamento de todos os portadores, para evitar que a ação patogênica cumulativa dos ovos do *S. mansoni* provoque alterações nos órgãos afetados, especialmente no fígado, resultando em hipertensão portal e outras formas graves da doença.

O praziquantel (PZQ) é o medicamento de primeira escolha para o tratamento de todos os tipos de esquistossomose. Desde a sua descoberta, em meados da década de 1970, a sua segurança e eficácia garantiram a sua utilização generalizada^25^, O tratamento com PZQ visa controlar a produção de ovos de esquistossomo, destruindo os vermes adultos, de modo que a morbidade resultante, incluindo complicações associadas e mortalidade, seja minimizada. No entanto, o tratamento não ajuda a reverter as complicações associadas à fibrose tecidual. Como o PZQ não é eficaz contra os estágios iniciais do esquistossomo imaturo e funciona exclusivamente contra vermes adultos, outras alternativas de tratamento devem ser consideradas nesses casos^25^. A detecção e o tratamento dos portadores objetivam também reduzir a expansão geográfica da esquistossomose. Uma das dificuldades para a detecção precoce dos portadores do *S. mansoni* é que a infecção pode evoluir de maneira silenciosa até a instalação das formas graves da doença.

A cura da esquistossomose não interrompe a progressão da doença glomerular em humanos ou em animais de experimentação. Esses dados podem sugerir que, apesar da importância dos antígenos esquistossomóticos no início da lesão glomerular, outros fatores, como a autoimunidade, podem levar ao desenvolvimento de anticorpos antinucleares (ANA), fator reumatoide e crioglobulinas, desempenhando um papel na evolução posterior das lesões^10^.

O tratamento com imunossupressão para a glomerulopatia associada à esquistossomose ainda é incerto. Martinelli et al. mencionam que não existe tratamento específico para as lesões glomerulares associadas à esquistossomose. As lesões de classe I regridem com o tratamento da infecção, e as lesões de classe II remitem com o tratamento antiesquistossômico (por exemplo, praziquantel) e com terapia antisalmonella (por exemplo, cotrimoxazol combinado, 960 mg duas vezes ao dia, e ampicilina 1 g a cada 6 h ou uma quinolona, como ciprofloxacina 1 g duas vezes ao dia). As classes III a V não respondem à erradicação de infecções, e corticosteroides ou agentes imunossupressores podem ser necessários^46^.

De acordo com os mesmos autores^47^, em um estudo realizado com 15 pacientes com diagnóstico de GESF que apresentavam a forma hepatoesplênica da esquistossomose mansônica, 10 indivíduos receberam tratamento imunossupressor. Sete foram classificados como resistentes à terapia, embora um paciente tenha se tornado responsivo aos esteroides durante o acompanhamento. Todos os três pacientes que responderam aos esteroides receberam simultaneamente ciclofosfamida para evitar recaídas frequentes. A terapia imunossupressora foi bem tolerada e sem relato de complicações.

Atualmente, não está claro qual é o papel da terapia imunossupressora na remissão desses pacientes. As diretrizes do KDIGO de 2021^45^ orientam que pacientes com infecção esquistossomótica e doença glomerular devem ser tratados com um agente antiparasitário apropriado, em dosagem e duração suficientes para erradicar o microrganismo. Não há indicações para o uso de agentes imunossupressores.

A dúvida quanto ao uso de imunossupressores na doença glomerular associada a essa infecção em especial está fortemente relacionada à incerteza de sua patogênese, especialmente nas formas membranoproliferativa e GESF. Contudo, a evolução para doença renal crônica, observada em 37,5% dos nossos pacientes com glomerulopatia associada à esquistossomose, em um tempo médio de acompanhamento de 5 anos [9], nos leva a questionar a necessidade de investigar melhor as vias patogênicas dessa doença, propor terapêuticas adequadas baseadas na patogênese e avaliar de forma mais precisa a evolução da doença renal.

## Conclusão

Em conclusão, o acometimento glomerular pela esquistossomose mansônica não tem sua patogênese completamente esclarecida e, por este motivo, ainda levanta dúvidas se somente o tratamento antiparasitário será suficiente para uma melhor sobrevida renal. É importante observar que muitos pacientes com glomerulopatia associada à esquistossomose provavelmente evoluirão para doença renal crônica. Essa probabilidade de um resultado renal desfavorável, associada à elevada prevalência dessa doença tropical, configura um grande desafio para a melhoria em saúde pública.

## Data Availability

Os dados utilizados neste estudo não estão disponíveis publicamente.
